# FT-Raman spectroscopic characterization of enamel surfaces irradiated with Nd:YAG and Er:YAG lasers

**DOI:** 10.15171/joddd.2016.033

**Published:** 2016-12-21

**Authors:** Sima Shahabi, Reza Fekrazad, Maryam Johari, Nasim Chiniforoush, Yashar Rezaei

**Affiliations:** ^1^Laser Research Center of Dentistry (LRCD), Tehran University of Medical Sciences, Tehran, Iran; ^2^Departmet of Biomaterials, School of Dentistry, Tehran University of Medical Sciences, Tehran, Iran; ^3^Laser Research Center in Medical Sciences (LRCMS), AJA University of Medical Sciences, Tehran, Iran; ^4^Faculty of Dentistry, Tabriz University of Medical Sciences, Tabriz, Iran

**Keywords:** Dental caries, Er:YAG lasers, Nd:YAG lasers, Raman spectroscopy

## Abstract

***Background.*** Despite recent advances in dental caries prevention, caries is common and remains a serious health problem. Laser irradiation is one of the most common methods in preventive measures in recent years. Raman spectroscopy technique is utilized to study the microcrystalline structure of dental enamel. In this study, FT-Raman spectroscopy was used to evaluate chemical changes in enamel structure irradiated with Nd:YAG and Er:YAG lasers.

***Methods.*** We used 15 freshly-extracted, non-carious, human molars that were treated as follows: No treatment was carried out in group A (control group); Group B was irradiated with Er:YAG laser for 10 seconds under air and water spray; and Group C was irradiated with Nd:YAG laser for 10 seconds under air and water spray. After treatment, the samples were analyzed by FT-Raman spectroscopy.

***Results.*** The carbonate content evaluation with regard to the integrated area under the curve (1065/960 cm^–1^) exhibited a significant reduction in its ratio in groups B and C. The organic content (2935/960 cm^-1^) area exhibited a significant decrease after laser irradiation in group B and C.

***Conclusion.*** The results showed that the mineral and organic matrices of enamel structure were affected by laser irradiation; therefore, it might be a suitable method for caries prevention.

## Introduction

Enamel structure of tooth is the hardest tissue in our body. It is a complex of mineral and organic material with 85% minerals, 12% water and 3% protein and lipid by volume. Hydroxyapatite is the mineral component of the enamel with hexagonal symmetry and the formula Ca_10_(PO_4_)_6_(OH)_2_.^1,2^ Except the trapped organic components, the main difference between hydroxyapatite and enamel structure’s apatite is the presence of about 3% CO_3_^2–^by weight, i.e. dental enamel apatite is carbonated hydroxyapatite.^3^ Despite recent advances in dental caries prevention, it is prevalent and remains a serious health problem. In addition, dental caries is considered the most prevalent disease during human life,^4-6^ with high prevalence in some individuals.^7^

Laser irradiations have been used for many types of treatment in dentistry, including removal of caries, inhibiting caries, tooth preparation for restorative dentistry, soft and hard tissue surgery, and for activation of dental bleaching agents.^8,9^ Since the 1960s, it has become increasingly apparent that high-power lasers can be used to decrease the rate of subsurface demineralization of enamel structure, changing its crystalline construction, solubility in various acids and its permeability.^10,11^ Enamel structure can be modified with laser irradiation treatment, causing surface roughness, cracks, fusion and formation of multiple pores and some bubble-like inclusions.^12,13^ A potential preventive effect of laser on healthy enamel structure has been demonstrated; the effect of irradiation on white spot lesions is still unclear.^14,15^ The mechanism(s) underlying caries prevention by laser irradiation treatment remain unclear; knowledge of the chemical composition of irradiated enamel may play a significant role in the field of caries prevention using various laser devices.

Raman spectroscopy is a method for studying the enamel structure;^16,17^ using Raman spectroscopy, the molecular vibrational bands of synthetic or biological materials can be identified.^18^ Although FT-Raman spectroscopic method is mostly compared with the better-known FTIR (Fourier transform infrared spectroscopy), the former has some advantages over the latter technique.^19^ With Raman spectra, there is little interference with water content of samples, making the technique appropriate for studying several biological samples.^20^ In this context, the aim of this study was to evaluate the chemical changes occurring in enamel structure irradiated with Nd:YAG and Er:YAG laser, using Raman spectroscopy analysis.

## Methods

### Collection and preparation of teeth

The teeth utilized in this study were collected based on the Ethics in Research Committee of the Dental School guidelines‏. Fifteen freshly extracted human molars were used. We did not use impacted teeth in the present study. After radiographic examination to make sure they were free from any erosion, cracks, caries, or any other defects, 0.1% thymol solution was used to store the teeth at 4°C until use. The required sample sizes were calculated according to previous studies.^21,22^ The crowns of the teeth were removed close to the cemento-enamel junction with double-faced diamond disks in a low-speed hand-piece (NSK Nakanishi Inc, Kanuma, Japan). Nail varnish was used to cover the buccal surface of each tooth, but for a 2×2-mm window on the enamel surface of the sample. The tooth samples were divided into 3 groups randomly (n=5).

### Experimental design

The sample surfaces were treated as follows: group A: no treatment; group B: Er:YAG laser irradiation; group C: Nd:YAG laser irradiation.

### Laser Irradiation

Group A (control group) was untreated. Specimens in group B were irradiated for 10 seconds with Er:YAG laser (US20D, DEKA, Italy) with the following settings: WL (wavelength) = 2,940 nm; Power = 0.5 W; PE (pulse energy) = 50 mJ; RR (repetition rate) = 10 Hz; PD (pulse duration) = 230 µs; irradiation was carried out 4 mm from the tooth surface, and was accompanied by water/air spray. Specimens in group C were irradiated for 10 seconds with Nd:YAG laser (Fotona, FIDELIS, Ljubljana, Slovenia), emitting energy at a wavelength of 1064 nm. The settings used were: power = 0.5 W; pulse duration=100 µsec; fiber 300 µm; irradiation was carried out 1 mm far from the enamel surface, with 300-µm fiber, in sweeping motion.^11,21^

After treatment, all the specimens were assessed by Raman spectroscopic analysis; all the specimens were also observed for any morphological changes, such as cracks or craters, under a stereomicroscope.

### FT-Raman spectroscopic analysis

The surfaces of the specimens were analyzed by Raman spectroscopy at two time intervals: before treatment and after the laser irradiation. Raman spectroscopy was carried out with a Bruker Senterra system (SENTERRA; Bruker Inc., Karlsruhe, Germany) using the 785- and 532-nm lasers. Alterations in mineral and organic enamel contents were analyzed by comparing the Raman peaks centered at 1071 cm^–1^ (p1) and 2940 cm^–1^(p2), to the peak at 961 cm^–1^ (p3). The areas of the Raman peaks were assessed with Graph 4.4 software (Ivan Johansen).

### Statistical analysis

SPSS 17 (SPSS Inc, Chicago, Ill., USA) was used to analyze the results. Paired samples t-test was used to analyze the alterations occurring after laser irradiations. A 95% confidence interval was applied to evaluate the statistical significance.

## Results

**Figure 1.**
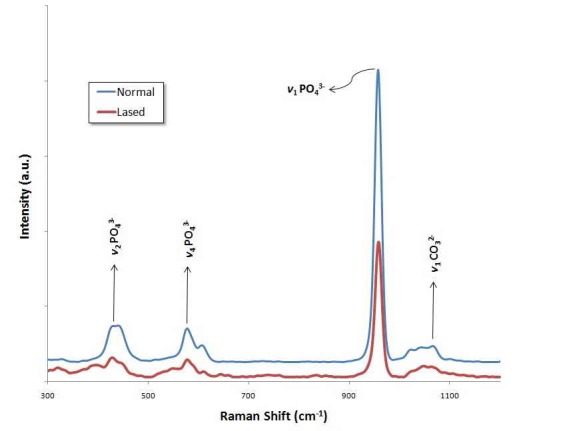
FT-Raman spectra of un-treated enamel surface (upper curve) and enamel surface lased with Nd:YAG laser (lower curve).

**Figure 2.**
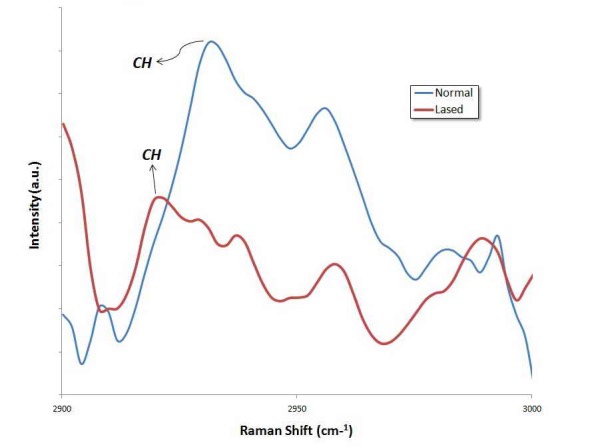
FT-Raman spectra of un-treated enamel surface (upper curve) and enamel surface lased with Er:YAG laser (lower curve).

**Figure 3.**
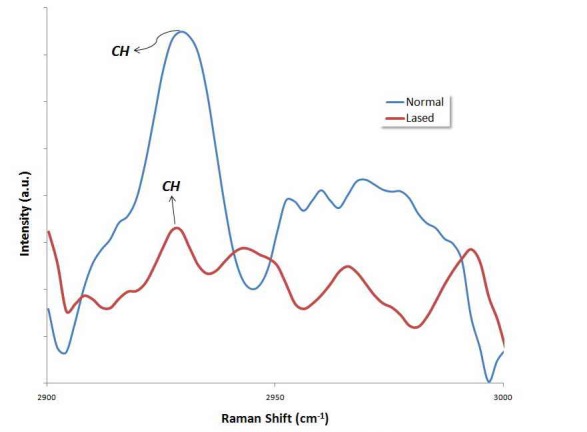
FT-Raman spectra of un-treated enamel surface (upper curve) and enamel surface lased with Nd:YAG laser (lower curve).

**Figure 4.**
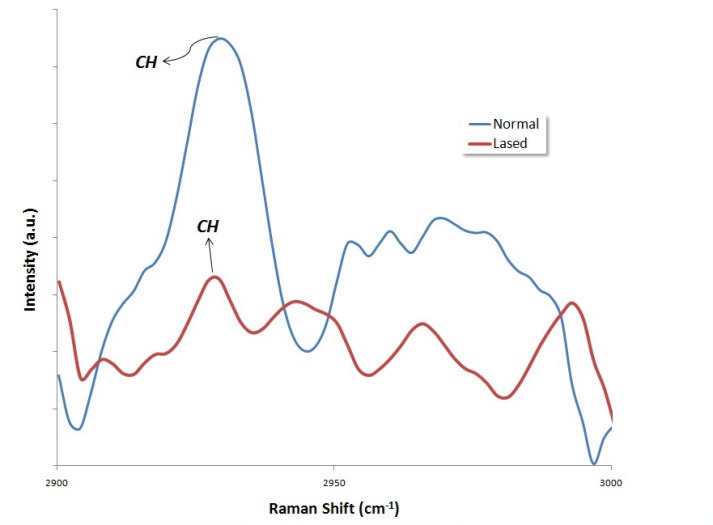
FT-Raman spectra of un-treated enamel surface (upper curve) and enamel surface lased with Er:YAG laser (lower curve)..

The FT-Raman spectra of the inorganic and organic ingredients of the dental enamel are displayed in Figures 1-4. The spectra of irradiated surface appear very homogeneous compared to those without any irradiation. Figures 1and2 depict the FT-Raman spectra of an untreated enamel surface, an enamel surface irradiated with Nd:YAG laser, and an enamel surface irradiated with Er:YAG laser, respectively, at a range of 300‒1200 cm^-1^. The most intense Raman peak, at 960 cm^-1^, is considered by the symmetrical stretching mode of PO_4_^3–^ groups in the mineral apatite component of enamel.^23,24^ The spectra showed a strong peak for PO_4_^3–^ in both normal and irradiated samples. The peak at 430 cm^–1^ and 591 cm^–1^ are subjected to the *ν*2 vibration of PO_4_^3–^ groups and *ν*4 vibration of PO_4_^3–^ groups.^24^ As might be seen in these figures, the mineral content, which is indicated by the intensity ratio of the peak at 960 cm^–1^ , decreased after laser irradiation. Although the peak integrated intensity of the phosphates content (960 cm^–1^) changed after laser treatment, the analysis showed no statistically significant decrease in the area ratio, either in the group irradiated with Nd:YAG laser (P =0.241) or in that irradiated with Er:YAG laser (P = 0.429).

The peak at 1065 cm^–1^ is attributed to the *ν*1 vibration of B-type carbonate (CO_3_^2−^) of the mineral.^25^ As may be seen, after irradiation with either Nd:YAG or Er:YAG lasers, the intensity of the peak at 1065 ± 10 cm^–1^decreased. The area ratios of intensity of CO_3_^2–^ peak to that of 960 cm^–1^ PO_4_^–3^ peak are listed in Table 1. Analysis of the area subjected to the carbonate content (1065/960 cm^–1^) depicted a significant decrease in the area ratio, both in specimens lased with Nd:YAG laser (P = 0.025) and in those irradiated with Er:YAG laser (P = 0.027). The results also revealed that the effect of Nd:YAG laser on the carbonate concentration was not significantly different from that of the Er:YAG laser (P = 0.196). Organic materials have vibrational bands at a range of 1200‒1700 cm^-1^; the relatively weak vibrational bands of amide I and amide III are in this range. CH_2_ stretching vibration produces a strong peak at 2935 ± 10 cm^-1^.^26^ Figures 3and4 illustrate the FT-Raman spectra of an un-treated enamel surface and those irradiated by the types of laser, at a range of 2900‒3000 cm^-1^. The CH_2_ vibrational bands (2935 ± 10 cm^-1^) lie in this range. As it can be seen in Figures 3and4, the intensity of CH_2_ band at 2935 ± 10 cm^-^1 is lower in the case of the irradiated surfaces, due to a considerable decrease in the concentration of organic materials.^27^ The analysis of the area subjected to organic content (2935/960 cm^-1^) exhibited a significant decrease in the area ratio after laser irradiation with either type of laser: Nd:YAG laser (P < 0.001) and Er:YAG laser (P = 0.003). These results also reveal that the effect of Nd:YAG laser on the intensity of the CH_2_ band at 2935 ± 10 cm^-1^was not significantly different from that of the Er:YAG laser (P = 0.350).

**Table 1:** Nd:YAG and Er:YAG laser effect on the mean and standard deviation of carbonate/PO_4_ and organic/PO_4_ content in natural enamel

**Bands**	**Integrated area ratio**					
	**Mean** **(un-lased)**	**Mean** **(lased via Nd:YAG)**	**P** ^a^	**Mean (un-lased)**	**Mean** **(lased via Er:YAG)**	**P**
**Carbonate/PO**_4_	0.069 (0.017)	0.048 (0.009)	0.025	0.060 (0.011)	0.049 (0.008)	0.027
**Organic/PO**_4_	0.101 (0.010)	0.051 (0.001)	< 0.001	0.107 (0.019)	0.047 (0.007)	0.003

^a^ All the data analyzed by paired-samples t-test.

## Discussion

The 2935 ± 10 cm^-1^ band has been used in a number of studies to semi-quantify organic changes, although this band is both clearer and stronger than the amides bands.^10,11,26^ The organic peaks at 1200–1700 cm^-1^ show broader properties because many materials may remain in partially amorphous and a hybrid phase.^25^ Dental enamel structure contains very low concentrations (1%) of organic matrix. Organic materials might have an important role in regulating the diffusion pathway in dental enamel, and thus have a great potential in prevention of caries with laser application.^11^ In all cases, the spectrum of a specimen before treatment was very similar to that of the same specimen after laser treatment, revealing that laser irradiation has only little effect on the enamel apatite and inflicted no serious damage on enamel structure. These findings show that laser irradiation does not have any effect on the crystal structure of tooth enamel.

The role of laser irradiation in caries prevention has been widely studied, using different lasers and deferent wavelengths, focusing on the enhancement of caries resistance caused by a decrease in the rate of enamel demineralization.^22,28-30^, Recently, many researchers studied the chemical analysis of enamel structure lased with various laser devices, using Raman spectroscopy. The results of these studies showed a decrease in the carbonate content, which enhances the acid resistance of enamel.^21,31^

Considering the chemistry underlying dental caries, one theory explaining the preservation of the subsurface area in caries zone is the low carbonate content of enamel since carbonate is thought to have a destabilizing effect.^32^ Our study showed that the concentration of CO_3_^-2^ decreased significantly after irradiating the enamel surface with Nd:YAG or Er:YAG laser. A loss of enamel carbonate content has been reported after CO_2_ and argon laser irradiation.^11,15,33^

Although the organic matrix accounts for less than 1% of enamel structure, it might have an important role in controlling enamel diffusion rate. The 2940 cm^-1^ band has been subjected to semi-quantification of organic changes since it is clearer than amides bands.^10,29^ In our study, a decrease in the band intensity was noticed after irradiating the enamel surface with either Nd:YAG or Er:YAG laser, indicating a reduction in the organic matrix. These results have been confirmed with other reports.^30,34^ It has been concluded that laser irradiation might afford changes and decomposition of both the organic matrix and the carbonated enamel structure’s apatite. As a result, it might have a great role in preventing enamel diffusion and reducing its dissolution, thus preventing enamel caries.

## Conclusion

The significant reduction of carbonate and organic matrix that occurs after applying Nd:YAG or Er:YAG lasers might indicate that this treatment is a suitable strategy for caries prevention. The results show that the Raman technique might be appropriate to survey changes in composition and structure of irradiated enamel.

## Acknowledgments

The authors would like to thank the staff at the Dental Laser Research Center of Dentistry (LRCD) at Tehran University of Medical Sciences for their assistance in carrying out the study.

## Authors’ contributions

SS, RF and MJ contributed to the concept and design of the study. SS, MJ, NC and YR contributed to data acquisition and interpretation, and drafted the manuscript. SS, MJ and YR contributed to critically revising the manuscript. All the authors have read and approved the final manuscript.

## Funding

This study was supported and funded by Dental Laser Research Center of Dentistry (LRCD) at Tehran University of Medical Sciences (grant No: 11818).

## Competing interests

The authors declare no competing interests with regards to the authorship and/or publication of this article.

## Ethics approval

The study protocol was approved by the Ethics Committee of Tehran University of Medical Sciences.
